# Neural complexity EEG biomarkers of rapid and post-rapid ketamine effects in late-life treatment-resistant depression: a randomized control trial

**DOI:** 10.1038/s41386-023-01586-4

**Published:** 2023-04-19

**Authors:** Nicholas Murphy, Amanda J. F. Tamman, Marijn Lijffijt, Dania Amarneh, Sidra Iqbal, Alan Swann, Lynnette A. Averill, Brittany O’Brien, Sanjay J. Mathew

**Affiliations:** 1 Baylor College of Medicine, Menninger Department of Psychiatry and Behavioral Sciences, Houston, TX USA; 2 The Menninger Clinic, Houston, TX USA; 3 Michael E. DeBakey VA Medical Center, Houston, TX USA

**Keywords:** Predictive markers, Translational research

## Abstract

Ketamine is an effective intervention for treatment-resistant depression (TRD), including late-in-life (LL-TRD). The proposed mechanism of antidepressant effects of ketamine is a glutamatergic surge, which can be measured by electroencephalogram (EEG) gamma oscillations. Yet, non-linear EEG biomarkers of ketamine effects such as neural complexity are needed to capture broader systemic effects, represent the level of organization of synaptic communication, and elucidate mechanisms of action for treatment responders. In a secondary analysis of a randomized control trial, we investigated two EEG neural complexity markers (Lempel-Ziv complexity [LZC] and multiscale entropy [MSE]) of rapid (baseline to 240 min) and post-rapid ketamine (24 h and 7 days) effects after one 40-min infusion of IV ketamine or midazolam (active control) in 33 military veterans with LL-TRD. We also studied the relationship between complexity and Montgomery-Åsberg Depression Rating Scale score change at 7 days post-infusion. We found that LZC and MSE both increased 30 min post-infusion, with effects not localized to a single timescale for MSE. Post-rapid effects of reduced complexity with ketamine were observed for MSE. No relationship was observed between complexity and reduction in depressive symptoms. Our findings support the hypothesis that a single sub-anesthetic ketamine infusion has time-varying effects on system-wide contributions to the evoked glutamatergic surge in LL-TRD. Further, changes to complexity were observable outside the time-window previously shown for effects on gamma oscillations. These preliminary results have clinical implications in providing a functional marker of ketamine that is non-linear, amplitude-independent, and represents larger dynamic properties, providing strong advantages over linear measures in highlighting ketamine’s effects.

## Introduction

Treatment-resistant depression (TRD) is commonly defined as two or more failed antidepressant treatments of adequate dose and duration. This presents a considerable clinical challenge across the lifespan. It is well documented that depression across the life span is associated with poor maintenance of homeostasis in executive and affective control networks [1–3]. This has classically been treated via serotonergic modulation; however, consensus on the heterogeneity of depressive bio-typing [4, 5] has helped to guide the development of non-monoaminergic drugs.

The N-methyl-D-aspartate receptor (NMDAR) antagonist ketamine has shown considerable clinical efficacy for TRD over the last decade [6, 7]. A sub-anesthetic dose of ketamine generates an antidepressant effect by modulating the coordination of glutamatergic communication and the release of brain-derived neurotrophic factor (BDNF) and the activation of mammalian target of rapamycin (mTOR) in the frontal network [8]. The role of mTOR in ketamine’s antidepressant effects is supported by findings that rapamycin administration prolonged the antidepressant effects of ketamine [9]. This modulation occurs via the blockade of presynaptic NMDARs at the inter-neuronal level of pyramidal circuits [10], leading to a short-term alteration of excitation/inhibition (E/I) balance. From the perspective of the chronic stress pathology model, ketamine acts to restore communicative properties of the pyramidal neurons that enhance regulation and connectivity, which have previously been degraded by prolonged stress-induced glutamate excitotoxicity [11]. Predictive biomarker studies suggest that inter-individual differences in ketamine antidepressant response might be related to biological factors responsible for the extent of a glutamatergic surge [12–16]. Extent of this surge may enhance synaptic formation and strength within the pre-frontal cortex [17]. The antidepressant efficacy of ketamine in older adults is mixed [6, 18], potentially reflecting age-related changes in binding properties of NMDAR [19], disturbed BDNF function [20], and the dysregulation of glutamatergic and inflammatory processes [21]. These age-related differences may impact the ability of ketamine to induce a clinically meaningful shift in E/I balance.

Little is known about mechanisms of action for ketamine for adults experiencing TRD late in life (LL-TRD). In a recent study [22] we investigated the safety, efficacy, and neurophysiology associated with a range of doses of ketamine (0.1, 0.25, 0.5 mg/kg) administered to LL-TRD patients, relative to LL-TRD patients receiving a psychoactive control (0.03 mg/kg midazolam). We established that the commonly used dose of 0.5 mg/kg ketamine was safe to administer in LL-TRD patients and has the expected effect of depressive symptom reduction up to 7 days after a single infusion. Using electroencephalography (EEG) we observed rapid potentiation of oscillatory gamma power (30+ Hz), which subsided within 4 h and was not found to be further modulated in the post-rapid window of 1–7 days post-infusion. Further, we identified a trend for increased gamma potentiation during the infusion to be associated with greater reduction in Montgomery-Åsberg Depression Rating Scale (MADRS) score at 7 days post-infusion. Our EEG findings support reports of ketamine-induced gamma band potentiation [13–15]. However, unlike previous studies in younger adults, which observed meaningful gamma potentiation at intervals up to 24 h [13–15] post-infusion, our peak of potentiation was within the first hour of infusion and had completely subsided within 4 h. In all cases gamma potentiation had often normalized within several days of a single infusion, while the peak MADRS reduction generally occurs within 7 days. Thus, gamma potentiation is limited as a biomarker of ketamine metabolic effects in this age group, leaving an unmet need to develop biomarkers encapsulating the myriad effects of ketamine on intra- and inter-neuronal communication. Additionally, gamma is a linear measurement. The power of an oscillation at a given electrode is a linear representation of processes occurring in cortical space-time and, as such, doesn’t provide a measurement of the broader systemic foundations for the oscillation. However, oscillations alone provide insufficient information to determine the extent to which communicative richness has been improved within a given neural system.

One promising biomarker is signal complexity, which has previously been used to estimate regional and global effects of anesthetic-dose ketamine on consciousness [23–25]. Complexity estimates the capacity for information processing within a system, and can refer to the randomness (e.g., Lempel-Ziv complexity (LZC) [26]) or regularity (e.g., multiscale entropy (MSE) [27]) of synaptic patterns within a system, depending on the method. Complexity represents the capacity for information processing within a system, referencing the orderliness of a timeseries where deviations from perfect order represent increasingly more dynamic communication. These methods can inform our understanding of ketamine’s effects in LL-TRD as they represent the richness of neural connectivity. LZC and MSE have been successfully applied to the exploration of psychiatric disorders [28, 29] including depression symptoms [30–32] and predicts depression treatment response to mirtazapine with high accuracy [33]. Depression is associated with maladaptive and rigid cognitive patterns, such as rumination, passive acceptance, and self-blame [32, 34]. Such introspective and rigid methods of emotion regulation have previously been related with altered EEG complexity [35, 36]. Ketamine’s proposed mechanism of action is the enhancement of excitatory activity among pyramidal neurons, increasing functional connectivity [37], and by extension, complexity [24], potentially generating pro-cognitive effects including enhanced cognitive flexibility [38]. Higher baseline neural complexity may provide individuals with the crucial neural foundation to access ketamine’s mechanism of action. In support, ketamine administration has been associated with increased complexity in healthy individuals [39], indicating less uniform communication. In this secondary analysis of our previously reported clinical trial [22] we investigated the effects of ketamine on neural complexity in rapid (i.e., within 4 h) and post-rapid time window models of clinical effects, as well as the relationship between symptom reduction and patterns of complexity. We hypothesized that sub-anesthetic ketamine would increase complexity in rapid (0–4 h post-infusion) and post-rapid (1–7 days post-infusion) measurement windows, relative to an active control (midazolam).

## Participants and methods

The study was funded by US Department of Veterans Affairs and was monitored by a Data and Safety Monitoring Board. The original clinical trial was registered at ClinicalTrials.gov (NCT02556606). All procedures were approved by the Baylor College of Medicine IRB and the Michael E. DeBakey VA Medical Center Research and Development Committee. Materials and methods have been described in detail in previous publications [22, 40]. Here we focus on analytical methods specific to this secondary analysis. All subjects provided written informed consent before any study-related activities were conducted. All procedures took place the Michael E. DeBakey VA Medical Center in Houston, Texas.

### Subjects

Thirty-three US military veterans (55–72 years, mean 62 ± 5.6, 10 female [37%]) with TRD were enrolled and randomized in a double-blind trial to either ketamine (KET) (0.5 [*N* = 11], 0.25 [*N* = 5], 0.1 mg/kg [*N* = 4]) or midazolam (MID) (0.03 mg/kg [*N* = 13]) treatment conditions. TRD was defined as two failed antidepressant trials of adequate dose or duration. Four subjects were removed from further analysis (MID *N* = 2, KET 0.1 *N* = 2) due to insufficient numbers of accepted channels after pre-processing. All patients had Montgomery Asberg Depression Rating Scale (MADRS) symptom scores >27, had a minimum score of 25 on the Mini Mental State Exam (MMSE) and were psychotropic medication-free for at least 7 days prior to infusion. Detailed inclusion and exclusion criteria are provided in Supplementary Text 1.

### Study design and procedures

The clinical trial structure is depicted in Supplementary Figs. 1 and 2, and described further in Supplementary Text 2. Eligible participants participated in ten visits to assess clinical and physiological effects of ketamine relative to active control midazolam. All subjects arrived after an overnight fast. On the infusion day measurements were repeated to capture pharmacokinetic information (60 min pre-infusion, 30, 60, 120, 240 min post-infusion). Patients were randomized to one of four dose groups using a Bayesian Adaptive Randomization strategy [22]: KET (0.1 mg/kg, 0.25 mg/kg, 0.5 mg/kg), MID (0.03 mg/kg). Neurophysiological measurements were conducted at pre-infusion baseline, during the 40-min infusion (at 30 min post-start of infusion), 60 min, 120 min, 240 min, 24 h, and 7 days post-infusion.

### EEG procedures

The procedures for the EEG recording in the clinical trial involved resting state EEG being recorded for 2 min for each of eyes closed and eyes open, using Curry 7, a 64-channel quick-cap system, and a SynAmps2 amplifier. In this analysis, we focused on the eyes closed recording to limit the influence of visual processing on signal variability and fluctuating attention due to wandering gaze. EEG was digitized at 1000 Hz, and impedance was kept below 10 kΩ. Data analysis and feature extraction was performed using custom Matlab scripts and routines adapted from the EEGLab toolbox [41], LZC procedures [42, 43], and multiscale entropy procedures [27]. Resting state EEG pre-processing procedures varied slightly from [22] to reflect the sensitivity of non-linear/complexity measures to non-cortical sources of activity/noise.

Resting state signals were filtered using independent high-pass (1 Hz) and low-pass (50 Hz) filters and resampled to 250 Hz. Line noise (60 Hz) and its harmonics (120, 180, 240 Hz) were removed using the Cleanline plugin [44]. Filtered data were then put into the artifact subspace reconstruction algorithm (ASR) [44] to handle bad channel detection and the removal of temporally sporadic artifacts (e.g., sudden bursts of movement, short periods of amplitude potentiation and other impedance related artifacts). ASR identified bad channels as having a scalp-wide correlation coefficient of <0.85, and applied reconstruction to burst, flat-line, and trending artifacts. Bad channels were reconstructed using spherical spline interpolation [41], and the EEG was converted to common average reference. Independent components were estimated using FASTICA [45] with principal components reduction equal to the rank of the data matrix. Ocular motion, muscular activity, and electrode noise were identified and removed using routines adapted from the MARA [46, 47] and TESA [48, 49] toolboxes. Because of the sensitivity of non-linear measures to noise features, additional spatial filtering was performed using Laplacian transformation to attenuate the influence of broad spatial artifacts on the data.

To reduce the number of statistical comparisons, and to focus on a generic cortical impression of complexity, the LZC and MSE were estimated first at the level of a single channel and then reduced to a single dimension by taking the mean across channels (e.g., [50]) (see Supplementary Fig. 3).

### Lempel-Ziv complexity

Lempel-Ziv complexity (LZC) is a measurement of the distance from uniformity of a time-series of finite length [43]. The degree of randomness is equivalent to the number of unique combinations of “0”s and “1”s that can be estimated within the signal when scanned left to right. To normalize the value between 0 and 1 we performed the LZC algorithm using a randomly shuffled version of the original EEG signal (surrogate) which represents the most complex (random) signal. LZC is equal to the number of unique combinations in the original signal divided by the number of unique combinations in the surrogate signal. An example of LZC coding is provided in Supplementary Text 3. To estimate LZC complexity we extracted the first 110 s of the RS EEG for each subject, restricting the data to eyes closed state. The first 10 s were excluded to reduce noise associated with boundary and edge effects, providing an analysis window of 100 s (25,000 samples). To facilitate the identification of patterns in the time-series the data were Hilbert transformed and binarized according to whether an individual value was greater than (1) or less than (0) the mean of the rectified signal [51].

### Multiscale entropy

MSE is a method of estimating the complexity of a signal as a function of different timescales [27, 52], representing the variability in different biological processes. Brain signals are transmitted within different spatial and temporal scales. MSE measures brain signal variability (transient temporal changes in neural signal) and describes signal regularity across a range of temporal scales from short (e.g., 2 ms intervals) to long (e.g., 40 ms intervals [52, 53]). The inclusion of different timescales removes the assumption that functional contributors to the regularity of the signal operate uniformly. This is achieved by applying coarse graining procedure to the finite length signal to achieve “X” number of signals equal to “Y” number of time-scales. Sample entropy is then estimated for each timescale. The complete mathematical rationale for MSE and its functional interpretation in biological signals is described in [52]. MSE was estimated on the same segments extracted for the LZC analysis. Due to the sensitivity of sample entropy to signal length we computed MSE on non-overlapping 4 s epochs and averaged across the epochs to achieve the final MSE estimate [50]. MSE was estimated using 20 scale factors, *m* of 2, and *r* of 0.5.

### Data analysis

Demographic and clinical characteristics were reported in our manuscript describing the primary endpoint [22], and are summarized for this sample in Table 1. We conducted the main statistical analyses for the complexity data across ketamine doses and conducted post hoc analysis examining main effects of dose. LZC and MSE were analyzed with respect to time and group in two contexts. Context 1 focused on rapid effects of KET relative to MID occurring from pre-infusion to 240 min post-infusion. Context 2 evaluated post-rapid effects of KET relative to MID occurring across pre-infusion, 24 h post-infusion, and 7 days post-infusion. To investigate the relationship between complexity and MADRS depression score, we examined correlations between baseline and potentiated complexity with day 7 changes in MADRS score. MADRS change at day 7 was selected as we adhered to our pre-specified protocol which identified day 7 as the primary clinical endpoint to reflect a clinically meaningful and enduring effect (see [22]). Each context used linear mixed models (LMM) to evaluate the contribution of time, drug, and scale (MSE only) to complexity. LMMs were run for full factorial models and re-run with non-significant fixed factors. The final evaluation of each context used the model with the lowest Bayes’ Information Criterion (BIC), which indicated the model with the greatest information content. All LMMs assumed random intercepts for each subject. In Supplementary Text 3, we describe post hoc analyses of the effects of KET dose group on complexity.

**Table 1:** Patient demographics.

	Midazolam *n* (%) or *M* (SD)	Ketamine (mg/kg) *n* (%) or *M* (SD)			
	0.03 mg/kg (*n* = 11)	0.1 (*n* = 2)	0.25 (*n* = 5)	0.5 (*n* = 11)	Ketamine all dosages
Demographic/health information					
Age (years)	63 (5.6)	69.5 (3.5)	62.0 (5.96)	61.0 (1.5)	62.22 (5.55)
Male	7 (63.6%)	2 (100%)	2 (40.0%)	8 (72.7%)	12 (66.7%)
White	6 (54.5%)	1 (50.0%)	2 (40.0%)	7 (63.6%)	10 (55.6%)
Non-Hispanic	10 (90.9%)	2 (100%)	4 (80%)	10 (90.9%)	16 (88.9%)
Weight (kg)	87.4 (14.9)	88.2 (6.6)	84.7 (6.9)	94.64 (1]8.7)	91.2 (15.5)
Baseline pre-infusion MADRS score	34.64 (4.34)	32.00 (4.24)	29.88 (3.96)	33.55 (3.21)	33.72 (3.39)

No significant differences observed between different dosage groups. Dose mixing effects are shown in the Supplementary Materials.

*M* mean, *SD* standard deviation.

### Correlations

Kendall’s tau correlations were performed to observe the relationship between pre-infusion baseline complexity and day 7 changes in MADRS score (day 7—Baseline), as well as the relationship between potentiated complexity (the percentage difference in complexity between baseline and 30 min, 24 h, 7 days) and day 7 changes in MADRS score. *p* values were corrected using Bonferroni adjustment within families of tests. To reduce the number of comparisons performed for MSE, timescales were reduced into four bins (scales 1–5, 6–10, 11–15, 16–20) and the average entropy within each bin was calculated.

## Results

Patient demographics are highlighted in Table 1. Rapid and post-rapid window outputs for LZC and MSE are described below. Detailed properties of each model’s fixed effects are shown in Supplementary Tables 1 and 2, and further description is provided in Supplementary Text 4.

### LZC—rapid effects model

**Fig. 1:**
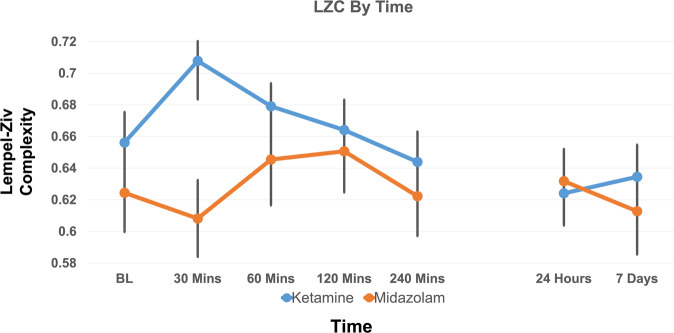
Line graph of predicted LZC. LZC is shown as a function of time post-infusion (rapid effects: baseline (BL) to 240 min, post-rapid effects 24 h to 7 days post-infusion). Black lines represent 95% confidence intervals.

We evaluated a model of LZC which had significant fixed effects of drug (*F* = 6.01, *p* = 0.018) and time × drug interaction (*F* = 3.84, *p* = 0.006) (see Fig. 1). The estimates of fixed effects indicated that LZC was broadly increased by KET relative to MID, which occurred most strongly during the 30 min post-infusion measurement.

### LZC—post-rapid effects model

Our mixed model revealed no significant fixed factors (drug, *F* = 0.095, *p* = 0.76; time, *F* = 1.12, *p* = 0.34) or interactions (drug × time, *F* = 0.23, *p* = 0.8) for the post-rapid time-window.

### MSE—rapid effects model

**Fig. 2:**
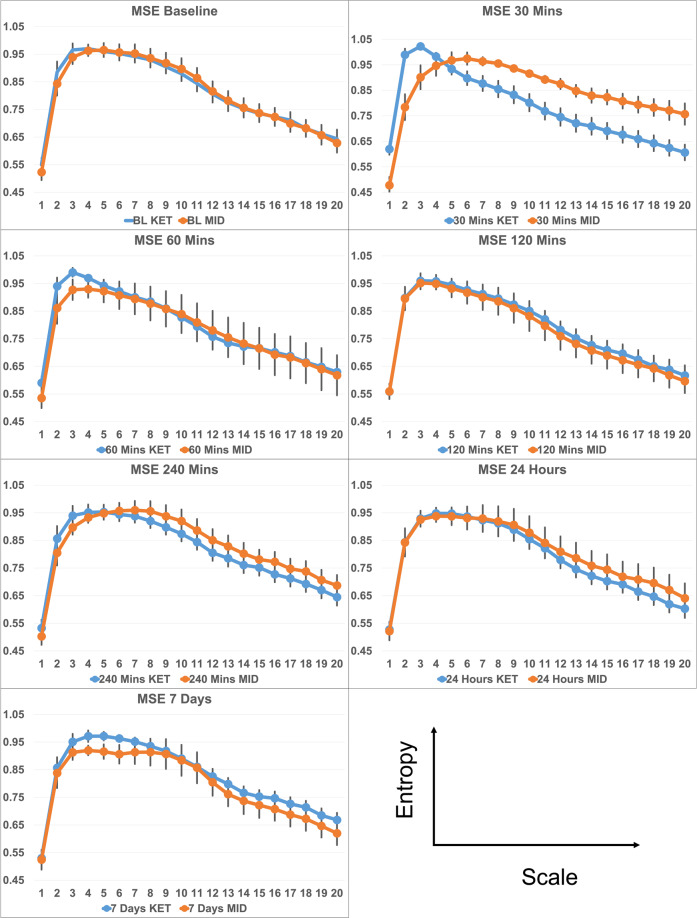
Line graph of predicted MSE. MSE is shown as a function of scale entropy (*Y* axis), timescale (*X* axis), and drug. Each graph represents a different time point. Black lines represent 95% confidence intervals.

We evaluated a model of MSE with significant fixed effects of drug (*F* = 9.2, *p* = 0.003), time (*F* = 11.68, *p* < 0.001), scale (*F* = 170.2, *p* = < 0.001), drug × time interaction (*F* = 13.85, *p* < 0.001), and drug × scale interaction (*F* = 3.4, *p* < 0.001) (see Fig. 2). Both treatment groups had MSE curves with increasing entropy toward the end of the first quarter of the total timescales which then declined toward scale 20. The effect of drug shows a broad reduction of MSE as a result of KET during day 1 relative to MID (note: day 1 consists of baseline and 30, 60, 120-, and 240-min post-infusion). The interaction terms denote that there are scale and time sensitive effects of KET, with the greatest drug effects occurring at 30 min post-infusion, and primarily affecting timescales 6–20.

### MSE—post-rapid effects model

The post-rapid effects model had significant fixed effects of day (*F* = 5.67, *p* = 0.004), drug (*F* = 4.77, *p* = 0.03), Scale (*F* = 144.98, *p* < 0.001), and a significant day × drug interaction (*F* = 7.85, *p* < 0.001). MSE for both groups, at each time point, followed the same pattern of changes across timescales as the rapid effects model. MSE was broadly reduced at 24 h relative to day 1 (*p* = 0.009) and day 7 (*p* = 0.013). However, day 1 and day 7 entropy values were not distinct. KET increased overall entropy relative to MID (*p* = 0.031). Simple effects analysis of the interaction effect confirmed that the distinction between day 1 and 24 h, and 24 h and 7 respectively, were exclusive to KET.

### Correlations

No significant correlations were observed using baseline or potentiated complexity for the relationship between pre-infusion baseline complexity and day 7 changes in MADRS score nor between potentiated complexity and day 7 changes in MADRS score.

## Discussion

In this secondary analysis of ketamine for late-life, TRD, we investigated rapid and post-rapid changes in neural complexity following a single infusion of IV ketamine. Additionally, we studied the relationship between complexity dynamics and MADRS score change at 7 days post-infusion as a marker of non-linear foundations for optimal clinical response. Two complementary measures of non-linear neural dynamics were used to measure complexity: LZC and MSE.

Rapid effects of ketamine involved increased LZC and MSE ~30 min post-initiation of infusion. Interaction modeling revealed that MSE changes were not localized to a single timescale and indicated that ketamine is associated with differential effects across the scope of functional contributors to shifting E/I balance. Post-rapid effects were restricted to MSE, with complexity reduced at 24 h across multiple timescales. However, effects reverted to baseline levels at day 7. Our findings support the hypothesis that a single sub-anesthetic ketamine infusion has time-varying effects on system-wide contributions to the evoked glutamatergic surge in individuals with LL-TRD. Further, we demonstrate that changes to complexity are observable outside of the time-window previously shown for effects on gamma oscillations. We are unable to determine the cause for differences in the post-rapid effects between LZC and MSE in our preliminary study but possibly reflect the differences in the physiological content of the two complexity measures. LZC is a measure of randomness; the complexity is equivalent to the number of patterns identifiable, and repeated, within the mixed signal. This approach treats complexity more as a blunt instrument, and is a suitable method for estimating the overall extent to which the system deviates from uniformity. We can consider this much the same as what event related potentials (ERP) represent relative to the Fourier transform. On the other hand, MSE demonstrates the regularity of information processing across different states of organization, or rather timescales. The choice to measure complexity with respect to time is inspired by the organization of oscillatory activity, where different functional processes have distinct temporal signatures. For example, top-down inhibition of the visual cortex operates at the frequency range of the alpha band [54], whereas local inhibition of microcircuits during a working memory task operate in the gamma band range [55]. With respect to function [27, 52], describe the different timescales of MSE as representing different cardiac processes that make up the structure of the heartbeat. In the context of the brain we can think of this as compartmentalizing the complexity of a system by its subsystems. Thus, the choice to use both LZC and MSE was so that we can identify with greater integrity the systemic effects of KET in late life depression. It is thus entirely possible that there could be discrepancies between LZC and MSE due to their inherent differences with regard to how information about neural activity is presented.

Single infusion studies have been the primary tool for mapping the clinical mechanism of action for ketamine treatment for TRD. EEG applications in this context have mainly focused on gamma oscillations as a proxy for pyramidal E/I balance [13–15]. Ketamine research has faced the challenge of identifying biological properties that form the ideal neural architecture for antidepressant effects. In recent work, the interaction between low baseline and ketamine-induced gamma oscillations has shown promise as a model of response likelihood [15]. A requirement for low initial gamma power and high ketamine-induced potentiation of gamma power implies that the optimal response is associated with a transformation from a low to high information state. Non-linear measurements such as complexity allow us to interpret the information transfer underpinning changes at the pyramidal level. Complexity provides us with insight into the functional adaptability and broad synaptic structure of the brain. By applying complexity measures to the study of ketamine’s mechanism of action, we show that we can robustly measure the expected effects on the rate and extent of communication within a given population of neurons (e.g., uniform to complex, low to high synaptic density).

To elucidate whether complexity could provide a measure of treatment response, we investigated the relationship between both baseline and potentiated complexity and treatment outcome at 7 days. Our findings suggest that while ketamine affects the level of complexity over time in our sample, the baseline complexity and the extent to which ketamine alters complexity does not directly impact antidepressant response. This leaves open the possibility that complexity may act in some complementary capacity to gamma oscillations, but that complexity itself is not a moderator or mediator of MADRS reduction. Future study designs should consider this, giving sufficient attention to achieving the statistical power required to perform an appropriate mediation analysis.

### Limitations and future work

As a secondary analysis, this investigation has several limitations. First, the original study was primarily designed to investigate dose optimization, resulting in between-group differences in dose that were not the central focus of this secondary analysis. To address this limitation, we conducted the main statistical analyses for the complexity data across doses and conducted post hoc analysis examining main effects of dose. However, due to the Bayesian adaptive randomization strategy which resulted in very few participants being given ketamine at 0.1 or 0.25 mg/kg doses, this limited our power to examine complexity values and antidepressant response across these differing treatment arms. Our lack of findings of correlations between complexity values and their change and depression changes could be due to mixing lower dose ketamine in seven patients with the larger group of patients with typical sub-anesthetic doses (dose mixing effects are explored in the Supplementary Material). Future studies should investigate the relationship between ketamine, depression, and other physiological markers in larger samples. Second, this sample had a narrow age range. Future studies should examine patients in a cohort with more variability in age. Third, complexity allows us to study the richness and meaningfulness of a signal [42, 56]. However, determining at what point higher complexity represents randomness remains challenging since it is a continuous measure without known cutoffs. Additionally, considering the sample size, we were unable to analyze possible moderating effects of biological sex. Given evidence that magnetoencephalogram LZC varies as a function of biological sex, an important avenue for future work is to examine whether the relationship between ketamine administration and complexity values vary as a function of sex differences [57, 58]. Future work should also account for variations in functional states that might affect dynamics associated with complexity. For example, supports the likelihood of greater complexity during the eyes open state [59]. As increasing complexity represents a tendency toward greater desynchronization it is logical to conclude that the influx of information to the brain during a state of eyes open would reduce the uniformity of synaptic firing to account for spontaneous cognitive processing. However, because 64-channel EEG does not lend itself to accurate source localization there was no means of systematically accounting for the sources of variability in signal complexity. To fully realize the extent to which complexity can be adopted as a biomarker of antidepressant action the mechanical influences of state changes must be addressed. Finally, we used a psychoactive drug control group, which allows us to measure the degree of complexity associated with ketamine strictly within an LL-TRD group but also only within the context of how midazolam affects complexity. It is necessary to complement this analysis by contrasting LL-TRD with age-matched healthy controls and patients given a non-active placebo.

In addition, because our study was a secondary analysis, it was outside the scope of this study to investigate the psychological correlates of complexity. One such important psychological construct is dissociation. Dissociation may have an important role in the relationship between ketamine and altered complexity. Previous work in healthy subjects has shown that associations of ketamine with increased signal diversity correlate with altered states of consciousness [24], raising the possibility that acute increases in signal complexity are associated with drug dissociative effects but are unrelated to durable changes in depression that follow drug administration. Given that dissociative adverse effects are common [60], can be distressing or frightening, and may lead to premature treatment discontinuation, it is important for future work to identify whether complexity can provide a biomarker for individuals who will demonstrate this adverse effect in advance of treatment initiation.

## Conclusions

We present preliminary evidence to support neural complexity measures as biomarkers of rapid and post-rapid effects of ketamine in LL-TRD. Our findings identified ketamine-induced complexity alterations in isolation of depressive symptom reduction. Future work should emphasize the interplay between complexity and properties of oscillations (power and cross-frequency interactions). These limitations notwithstanding, our study is, to our knowledge, the first examination of the effect of ketamine on complexity in a clinically depressed sample. Our findings support the hypothesis that a single sub-anesthetic ketamine infusion has time-varying effects on system-wide contributions to the evoked glutamatergic surge in LL-TRD. Further, changes to complexity were observable outside the time-window previously shown for effects on gamma oscillations. These preliminary results have clinical implications in providing a functional marker of ketamine that is non-linear, amplitude-independent, and represents larger dynamic properties, providing strong advantages over linear measures in highlighting ketamine’s effects. To enhance clinical actionability of complexity as a biomarker, our findings should be replicated in a larger sample with multiple age cohorts and age-matched healthy controls. Conducting a larger study will allow a well-powered multivariate investigation of the physiological properties of ketamine induced MADRS reduction.

### Supplementary information

Supplementary text, table, and figures

Supplementary Table 2 – table of fixed effects of drug, time, and drug by time for Multiscale Entropy
