# Correction: Neural complexity EEG biomarkers of rapid and post-rapid ketamine effects in late-life treatment-resistant depression: a randomized control trial

**DOI:** 10.1038/s41386-023-01618-z

**Published:** 2023-06-22

**Authors:** Nicholas Murphy, Amanda J. F. Tamman, Marijn Lijffijt, Dania Amarneh, Sidra Iqbal, Alan Swann, Lynnette A. Averill, Brittany O’Brien, Sanjay J. Mathew

**Affiliations:** 1 Baylor College of Medicine, Menninger Department of Psychiatry and Behavioral Sciences, Houston, TX USA; 2 The Menninger Clinic, Houston, TX USA; 3 Michael E. DeBakey VA Medical Center, Houston, TX USA

**Keywords:** Predictive markers, Translational research

Correction to: *Neuropsychopharmacology* 10.1038/s41386-023-01586-4, published online 19 April 2023

The article “Neural complexity EEG biomarkers of rapid and post-rapid ketamine effects in late-life treatment-resistant depression: a randomized control trial”, written by Nicholas Murphy, Amanda J. F. Tamman, Marijn Lijffijt, Dania Amarneh, Sidra Iqbal, Alan Swann, Lynnette A. Averill, Brittany O’Brien, and Sanjay J. Mathew was originally published electronically on the publisher’s internet portal on 19 April without open access. With the author(s)’ decision to opt for Open Choice the copyright of the article changed on 24 May 2023 to © The Authors 2023 and the article is forthwith distributed under a Creative Commons Attribution 4.0 International License, which permits use, sharing, adaptation, distribution and reproduction in any medium or format, as long as you give appropriate credit to the original author(s) and the source, provide a link to the Creative Commons licence, and indicate if changes were made. The images or other third party material in this article are included in the article’s Creative Commons licence, unless indicated otherwise in a credit line to the material. If material is not included in the article’s Creative Commons licence and your intended use is not permitted by statutory regulation or exceeds the permitted use, you will need to obtain permission directly from the copyright holder. To view a copy of this licence, visit http://creativecommons.org/ licenses/by/4.0/. The original article has been corrected.

