# Supplementary material for: Neural complexity EEG biomarkers of rapid and post-rapid ketamine effects in late-life treatment-resistant depression: a randomized control trial
Source: Neuropsychopharmacology. 2023 Apr 19;48(11):1586–93. doi: 10.1038/s41386-023-01586-4 (PMC10516885; doi:10.1038/s41386-023-01586-4)
Supplement: Supplementary file 1 — Supplementary text, table, and figures [file 41386_2023_1586_MOESM1_ESM.docx]

**Supplementary Text 1 – Inclusion and exclusion criteria****

**Inclusion criteria**

- Age ≥55 years
- Current MDE (Unipolar) based on the MINI 7.0
- History of ≥1 previous episode of depression prior to the current episode (recurrent MDD) or chronic MDD (of at least two years' duration)
- Failure to respond to ≥ 2 adequate trials of FDA-approved antidepressants determined by the ATRQ criteria
- QIDS-SR ≥14
- MADRS ≥ 27
- CGI-S ≥ 4
- Able to understand and sign informed consent
- Had EEG data

**Exclusion criteria**

- Currently taking fluoxetine
- History of bipolar disorder, schizophrenia, schizoaffective disorder or any psychotic disorder
- Documented history of a psychotic disorder in a first-degree relative
- Current diagnosis of OCD or eating disorder
- Alcohol or substance use disorder (except nicotine) within the preceding 3 months
- Clinically significant personality disorder that would, in the investigator's judgment, preclude safe study participation
- Serious and imminent suicidal or homicidal risk
- Serious, unstable medical illnesses including respiratory [obstructive sleep apnea, or history of difficulty with airway management during previous anesthetics], cardiovascular [including ischemic heart disease and uncontrolled hypertension], and neurologic [including history of severe head injury]
- Clinically significant abnormal findings of laboratory parameters [including urine ECG, toxicology screen for drugs of abuse], physical examination, or ECG
- Hypertension (systolic BP > 160 mm Hg or diastolic BP > 90 mm Hg)
- Participants with one or more seizures without a clear and resolved etiology
- Participants starting hormonal treatment in the 3 months prior to Screening
- Past intolerance or hypersensitivity to ketamine, or history of recreational use of PCP or ketamine
- Past intolerance or hypersensitivity to midazolam
- MMSE < 25 at Screening, suggesting age-related cognitive decline or mild dementia
- Ongoing use of medications with known activity at the NMDA or AMPA glutamate receptor [e.g., riluzole, amantadine, lamotrigine, memantine, topiramate, dextromethorphan, D-cycloserine], or the mu-opioid receptor
- Ongoing use of the following medications: St John's Wort, theophylline, tramadol, metrizamide
- Decrease of >25% in depressive symptoms as reflected by the QIDS-SR score from Screening to Randomization
- ECT treatment within 6 months prior to Screening
- Current VNS or rTMS therapy
- Currently taking fluoxetine
- History of bipolar disorder, schizophrenia, schizoaffective disorder or any psychotic disorder
- Documented history of a psychotic disorder in a first-degree relative
- Current diagnosis of OCD or eating disorder
- Alcohol or substance use disorder (except nicotine) within the preceding 3 months
- Clinically significant personality disorder that would, in the investigator's judgment, preclude safe study participation
- Serious and imminent suicidal or homicidal risk
- Serious, unstable medical illnesses including respiratory [obstructive sleep apnea, or history of difficulty with airway management during previous anesthetics], cardiovascular [including ischemic heart disease and uncontrolled hypertension], and neurologic [including history of severe head injury]
- Clinically significant abnormal findings of laboratory parameters [including urine ECG, toxicology screen for drugs of abuse], physical examination, or ECG
- Hypertension (systolic BP > 160 mm Hg or diastolic BP > 90 mm Hg)
- Participants with one or more seizures without a clear and resolved etiology
- Participants starting hormonal treatment in the 3 months prior to Screening
- Past intolerance or hypersensitivity to ketamine, or history of recreational use of PCP or ketamine
- Past intolerance or hypersensitivity to midazolam
- MMSE < 25 at Screening, suggesting age-related cognitive decline or mild dementia
- Ongoing use of medications with known activity at the NMDA or AMPA glutamate receptor [e.g., riluzole, amantadine, lamotrigine, memantine, topiramate, dextromethorphan, D-cycloserine], or the mu-opioid receptor
- Ongoing use of the following medications: St John's Wort, theophylline, tramadol, metrizamide
- Decrease of >25% in depressive symptoms as reflected by the QIDS-SR score from Screening to Randomization
- ECT treatment within 6 months prior to Screening
- Current VNS or rTMS therapy

**Abbreviations: Mini-International Neuropsychiatric Interview (MINI); Antidepressant Treatment Response Questionnaire (ATRQ); Quick Inventory of Depressive Symptomatology-Self Report (QIDS-SR);Montgomery Asberg Depression Rating Scale (MADRS);Clinical Global Impression-Severity (CGI-S); Mini-Mental State Examination (MMSE); N-methyl-D-aspartate (NMDA); Alpha-Amino-3-Hydroxy-5-Methyl-4-Isoxazole Propionic Acid (AMPA); Electroconvulsive Therapy (ECT); Vagus nerve stimulation (VNS); Repetitive transcranial stimulation (rTMS).

**Supplementary figure 1– Clinical trial flow chart.**.

1:3 Randomization

Midazolam 0.03mg/kg

Midazolam 0.03mg/kg

Ketamine 0.10mg/kg

Adaptive randomization

Ketamine 0.25mg/kg

Ketamine 0.50mg/kg

**Supplementary Text 2: description of randomization, blinding, and intervention procedures**

**Randomization:** As shown in Supplementary figure 1, the initial randomization ratio was 1:3. We used a Bayesian Adaptive Randomization approach in which this randomization ratio shifts after allocating the first 20 participants and at pre-specific regular intervals thereafter that are designated based on Bayesian posterior probabilities. This approach uses these probabilities to allocate more participants to conditions that are showing greater promise. Decision rules based on a posterior probability of greater than 0.975 that the condition is better than the next best condition stopped allocation in Arm 2 to MID, KET 0.25 mg/kg and KET 0.1 mg/kg in favor of allocation to KET 0.5 mg/kg. Arm 1 remained open for allocation to MID. The study was stopped after thirty-three subjects were randomized. Further details can be found in [1].

**Intervention description:** The research pharmacist prepared the midazolam or ketamine IV medication in a 100ml NS bag, dissolving in a 0.9% saline solution in total volume of 100ml, in accordance with the patient’s weight and other clinically relevant factors. Dosage was kept consistent or adjusted over the treatment course for each individual patient to reach a dose at which a mild dissociative effect was reported [2]. The infusion was administered in a private room in a hospital with monitoring of vital signs by a board-certified anesthesiologist with an infusion pump at a constant rate.

The initial dose of ketamine was weight-based using a 0.50 mg/kg ketamine calculation and other clinically relevant factors. Dosage was maintained or adjusted over the course of treatment for each individual patient to reach a dose in which the patient reported a mild dissociative effect

**Supplementary figure 2.** Diagram of study randomization, design, and analysis.


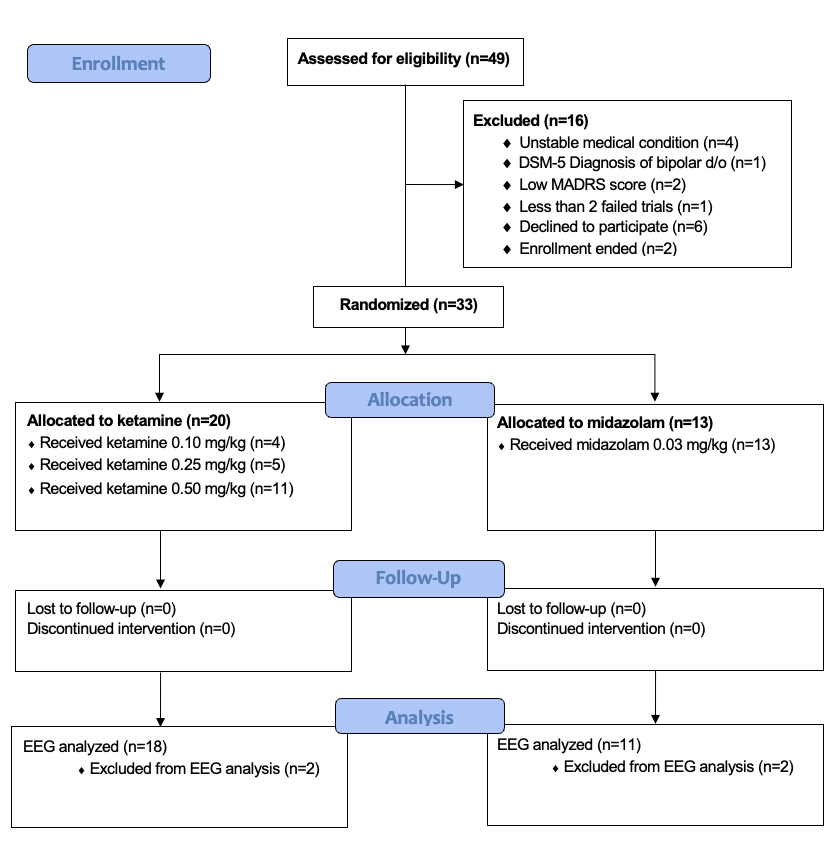


**Supplementary Text 3 – Further details about data analysis:**

1. **Filtering**

Cleanline is an adaptive filtering method which uses statistical thresholding to remove sinusoids that act as significant coefficients in a regression model of the requested line-noise frequencies. This overcomes the problem of non-specificity associated with traditional notch-filtering which can lead to the injection of time-domain distortions, and/or the failure to capture the true line-noise (which is often inconsistent with expectations and does not present as a single stationary sinusoid given a finite period of time [3].

1. **Spatial similarity (NPP cluster tests)**

To determine that dimension reduction would not remove important variability in the data we ran non-parametric permutation tests of the data at time 1 and time 2 between groups. This is a form of mass-univariate testing that uses time series (in our case the time scales) and spatial (channels) information to find clusters of significant activity. These clusters are then tested against a permuted version of the data (1000 permutations) which acts as the distribution for significance testing. Clusters are built by finding neighboring time points that meet criteria for significance (P<.05) (and test statistic with the same sign), and then looking to see if spatial neighbors also fit this pattern. This iterates on until we exhaust the data and can then identify unique spaces on the scalp where a chunk of consecutive time points all show a significant difference.

In supplementary figure 3 the binary maps show the clusters identified (one in each time point).  The numbers on the left are the indices of the channels, timescale is along the bottom. What we found was that in time 2 all electrodes are significantly greater MSE in the KET group for the first 4 timescales (positive cluster). Then timescales 6-20 are all significantly lower in the KET group for time 2 in almost all electrodes (negative cluster).

This indicates that dimension reduction is appropriate as it suggests that if we estimate MSE for all channels individually and then estimate the mean we have approximately the same signal across all channels.


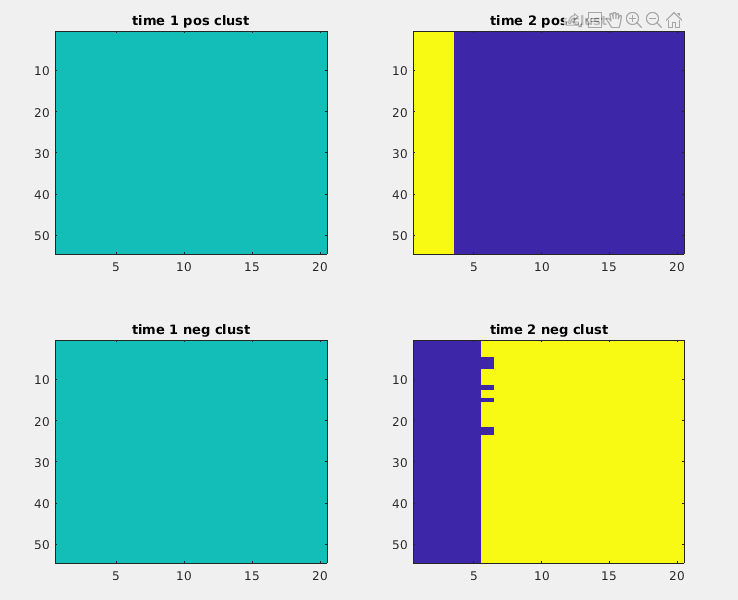


**Supplementary figure 3. Binary cluster maps.** Non-parametric permutation tests were used to derive cluster maps of (clockwise from top left) a) time 1 positive cluster, b) time 2 positive cluster, c) time 1 negative cluster, and d) time 2 negative cluster. We demonstrate the spatial similarity across time for complexity estimates using cluster-based non-parametric permutation analysis and our single-dimension approach. The identification by the cluster-based non-parametric permutation analysis of only a single scalp-wide cluster, when using spatial nearest neighbors with a maximum distance of 3.5 cm, indicates high spatial correlation and supports the reduction of the LZC and MSE channel data to single LZC and MSE dimensions.

|  | **Estimate (lower bound – upper bound)** | ***t*(df)** | ***p*-value** |
| --- | --- | --- | --- |
| **Rapid effects** | | | |
| **Main effect of drug** | | | |
| Ketamine† | 0.04(-0.02-0.09) | 1.38(96) | 0.17 |
| **Main effect of time** | | | |
| 30 minutes†† | -0.01(-0.06-0.02) | -1.09(114) | 0.28 |
| 60 minutes | 0.02(-0.02-0.06) | 0.86(114) | 0.39 |
| 120 minutes | 0.02(-0.01-0.06) | 1.27(114) | 0.21 |
| 240 minutes | -0.02(-0.05-0.02) | -0.85(114) | 0.40 |
| **Time*Drug interaction** | | | |
| 30 minutes*ketamine | 0.07(0.02-0.12) | 2.92(113) | 0.004 |
| 60 minutes*ketamine | 0.005(-0.05-0.06) | 0.19(113) | 0.85 |
| 120 minutes*ketamine | -0.02(-0.07-0.03) | -0.72(113) | 0.47 |
| 240 minutes*ketamine | 0.01(-0.04-0.06) | 0.40(113) | 0.69 |
| **Post-rapid effects** | | | |
| **Main effect of drug** | | | |
| Ketamine | 0.02 (-0.04-0.07) | 0.58(66) | 0.56 |
| **Main effect of time** | | | |
| 24 hours | -0.01(-0.05-0.03) | -0.51(52) | 0.61 |
| 7 days | -0.01(-0.05-0.04) | -0.30(52) | 0.77 |
| **Time*Drug interaction** | | | |
| 24 hours*ketamine | -0.02(-0.07-0.03) | -0.67(51) | 0.51 |
| 7 days*ketamine | -0.01(-0.06-0.04) | -0.36(51) | 0.72 |

**Supplementary Table 1:** table of fixed effects of drug, time, and drug by time for Lempel-Ziv Complexity

**Note**:

| † The reference variable for drug fixed effects estimates is midazolam |
| --- |
| †† The reference variable for time fixed effects estimates is baseline |

**Supplementary Text 4 – Further description of results**

1. **LMM model building**

***MSE – rapid effects:*** Our initial model indicated that scale*time and drug*scale*time interactions were not significantly contributing to the model (Schwarz’s Bayesian information criterion [SBIC] = -2988.48). Our second run removed these and reduced the Schwarz’s Bayesian information criterion (-4047.83) while not substantially changing the condition R^2^ estimation (model 1 = 0.64, model 2= 0.62).

***MSE – post-rapid effects:*** Our initial model indicated that scale*day and scale *drug were not significant contributors to the model (Schwarz’s Bayesian information criterion [SBIC] = -2314.57). Our second run removed these and reduced the Schwarz’s Bayesian information criterion (-2721.26) while not substantially changing the condition R^2^ estimation (model 1 = 0.688, model 2= 0.685).

1. **Dose Effects**
   To evaluate the contributions of dose to the variance in complexity we conducted a univariate ANOVA with a dependent variable of complexity and fixed effect of dose for each model, with Bonferonni corrected posthoc testing of the levels of the fixed effect.

   ***LZC - Rapid Effects****:* Follow-up evaluation of dose effects showed a significant effect of dose (F = 3.2, P = 0.025, η^2^ = .066) driven by increased LZC in the 0.5 KET group relative to the MID group (P = 0.025, CI= .004 : .084)).

   ***LZC - Post Rapid Effects****:* There were no significant fixed effects of dose for the post-rapid model (F = .63, P = .6, η^2^ = .024).

   ***MSE - Rapid Effects:*** There was a significant main effect of dose (F = 6.03, P<0.001, η^2^ = .006). Posthoc testing demonstrated a broad reduction of LZC in the 0.1 KET group (0.1 KET<0.25 KET, P<.001, CI= -.09 : -.01; 0.1 KET<0.5KET, P = 0.03, CI= -.07 : -.002; 0.1 KET<MID, P<.001, CI= -.08 : -.01).

   ***MSE - Post-Rapid Effects****:* Dose was not a significant main effect in the post-rapid effects model (F = 0.41, P = 0.74, η^2^ = .001).

**References**

1. O’Brien B, Green CE, Al-Jurdi R, Chang L, Lijffijt M, Iqbal S, et al. Bayesian adaptive randomization trial of intravenous ketamine for veterans with late-life, treatment-resistant depression. Contemp Clin Trials Commun. 2019;16:100432.

2. Pennybaker SJ, Niciu MJ, Luckenbaugh DA, Zarate CA. Symptomatology and predictors of antidepressant efficacy in extended responders to a single ketamine infusion. J Affect Disord. 2017;208:560–566.

3. Bigdely-Shamlo N, Mullen T, Kothe C, Su K-M, Robbins KA. The PREP pipeline: standardized preprocessing for large-scale EEG analysis. Front Neuroinformatics. 2015;9:16.
