# Supplementary material for: Neural complexity EEG biomarkers of rapid and post-rapid ketamine effects in late-life treatment-resistant depression: a randomized control trial
Source: Neuropsychopharmacology. 2023 Apr 19;48(11):1586–93. doi: 10.1038/s41386-023-01586-4 (PMC10516885; doi:10.1038/s41386-023-01586-4)
Supplement: Supplementary file 2 — Supplementary Table 2 – table of fixed effects of drug, time, and drug by time for Multiscale Entropy [file 41386_2023_1586_MOESM2_ESM.docx]

|  | **Estimate (lower bound – upper bound)** | | | | | ***t*(df)** | ***p*-value** |
| --- | --- | --- | --- | --- | --- | --- | --- |
| **Rapid effects** | | | | | | | |
| **Main effect of drug** | | | | | | | |
| Ketamine† | 0.03(-0.02-0.08) | | | | | 1.18(618) | 0.239 |
| **Main effect of time** | | | | | | | |
| 30 minutes†† | 0.05(0.03-0.07) | | | | | 4.31(2752) | <.001 |
| 60 minutes | -0.02(-0.04-0.01) | | | | | -1.34(2752) | 0.181 |
| 120 minutes | -0.04(-0.06-0.01) | | | | | -3.35(2752) | 0.001 |
| 240 minutes | 0.02(0.00-0.04) | | | | | 1.77(2752) | 0.078 |
| **Time*drug interaction** | | | | | | | |
| 30 minutes*ketamine | -0.08(-0.10--0.05) | | | | | -5.57(2752) | <.001 |
| 60 minutes*ketamine | 0.00(-0.03-0.03) | | | | | 0.03(2752) | 0.979 |
| 120 minutes*ketamine | 0.01(-0.01-0.04) | | | | | 1.09(2752) | 0.275 |
| 240 minutes*ketamine | -0.02(-0.05-0.00) | | | | | -1.58(2752) | 0.113 |
| **Main effect of scale** |  | | |  |  |  |  |
| 2 | 0.32(0.28-0.36) | | | | | 15.38(2752) | <.001 |
| 3 | 0.40(0.36-0.44) | | | | | 19.50(2752) | <.001 |
| 4 | 0.42(0.38-0.46) | | | | | 20.51(2752) | <.001 |
| 5 | 0.43(0.39-0.47) | | | | | 20.65(2752) | <.001 |
| 6 | 0.42(0.38-0.46) | | | | | 20.44(2752) | <.001 |
| 7 | 0.41(0.37-0.45) | | | | | 20.04(2752) | <.001 |
| 8 | 0.40(0.36-0.44) | | | | | 19.47(2752) | <.001 |
| 9 | 0.38(0.34-0.42) | | | | | 18.50(2752) | <.001 |
| 10 | 0.36(0.32-0.40) | | | | | 17.47(2752) | <.001 |
| 11 | 0.33(0.29-0.37) | | | | | 15.95(2752) | <.001 |
| 12 | 0.30(0.26-0.34) | | | | | 14.30(2752) | <.001 |
| 13 | 0.27(0.23-0.31) | | | | | 12.98(2752) | <.001 |
| 14 | 0.25(0.20-0.29) | | | | | 11.83(2752) | <.001 |
| 15 | 0.23(0.19-0.27) | | | | | 11.03(2752) | <.001 |
| 16 | 0.21(0.17-0.25) | | | | | 10.30(2752) | <.001 |
| 17 | 0.20(0.15-0.24) | | | | | 9.41(2752) | <.001 |
| 18 | 0.18(0.14-0.22) | | | | | 8.73(2752) | <.001 |
| 19 | 0.16(0.12-0.20) | | | | | 7.60(2752) | <.001 |
| 20 | 0.14(0.10-0.18) | | | | | 6.58(2752) | <.001 |
| **Scale*drug interaction** | | | | | | | |
| 2*ketamine | 0.32(-0.03-0.08) | | | | | 0.97(2752) | 0.332 |
| 3*ketamine | 0.40(-0.05-0.05) | | | | | 0.10(2752) | 0.921 |
| 4*ketamine | 0.42(-0.08-0.03) | | | | | -1.01(2752) | 0.311 |
| 5*ketamine | 0.43(-0.10-0.00) | | | | | -1.86(2752) | 0.063 |
| 6*ketamine | 0.42(-0.11--0.01) | | | | | -2.38(2752) | 0.017 |
| 7*ketamine | 0.41(-0.12--0.02) | | | | | -2.64(2752) | 0.008 |
| 8*ketamine | 0.40(-0.13--0.02) | | | | | -2.83(2752) | 0.005 |
| 9*ketamine | 0.38(-0.13--0.03) | | | | | -2.96(2752) | 0.003 |
| 10*ketamine | 0.36(-0.14--0.03) | | | | | -3.21(2752) | 0.001 |
| 11*ketamine | 0.33(-0.14--0.03) | | | | | -3.28(2752) | 0.001 |
| 12*ketamine | 0.30(-0.14--0.04) | | | | | -3.34(2752) | 0.001 |
| 13*ketamine | 0.27(-0.14--0.04) | | | | | -3.29(2752) | 0.001 |
| 14*ketamine | 0.25(-0.13--0.03) | | | | | -3.12(2752) | 0.002 |
| 15*ketamine | 0.23(-0.13--0.03) | | | | | -3.01(2752) | 0.003 |
| 16*ketamine | 0.21(-0.13--0.03) | | | | | -3.04(2752) | 0.002 |
| 17*ketamine | 0.20(-0.13--0.03) | | | | | -2.96(2752) | 0.003 |
| 18*ketamine | 0.18(-0.14--0.04) | | | | | -3.30(2752) | 0.001 |
| 19*ketamine | 0.16(-0.13--0.03) | | | | | -3.12(2752) | 0.002 |
| 20*ketamine | 0.14(-0.13--0.03) | | | | | -3.09(2752) | 0.002 |
|  | |  | **Post-rapid effects** | | | | |
| **Main effect of drug** | | | | | | | |
| Ketamine | 0.030(-0.008-0.068) | | | | | 1.582 | 0.068 |
| **Main effect of time** | | | | | | | |
| 24 hours | -0.018(-0.038-0.002) | | | | | -1.809 | 0.002 |
| 7 days | -0.012(-0.031-0.008) | | | | | -1.181 | 0.008 |
| **Time*drug interaction** | | | | | | | |
| 24 hours*ketamine | -0.013(-0.038-0.011) | | | | | -1.090 | 0.011 |
| 7 days*ketamine | 0.038(0.012-0.063) | | | | | 2.935 | 0.063 |
| **Main effect of scale** |  | | |  |  |  |  |
| 2 | 0.322(0.29-0.35) | | | | | -1.809 | 0.35 |
| 3 | 0.410(0.38-0.44 | | | | | -1.181 | 0.44 |
| 4 | 0.427(0.40-0.46) | | | | | -1.809 | 0.46 |
| 5 | 0.427(0.40-0.46) | | | | | -1.181 | 0.46 |
| 6 | 0.420(0.39-0.45) | | | | | -1.809 | 0.45 |
| 7 | 0.414(0.38-0.44) | | | | | -1.181 | 0.44 |
| 8 | 0.402(0.37-0.43) | | | | | -1.809 | 0.43 |
| 9 | 0.383(0.35-0.41) | | | | | -1.181 | 0.41 |
| 10 | 0.356(0.33-0.39) | | | | | -1.809 | 0.39 |
| 11 | 0.322(0.29-0.35) | | | | | -1.181 | 0.35 |
| 12 | 0.281(0.25-0.31) | | | | | -1.809 | 0.31 |
| 13 | 0.249(0.22-0.28) | | | | | -1.181 | 0.28 |
| 14 | 0.224(0.19-0.25) | | | | | -1.809 | 0.25 |
| 15 | 0.207(0.18-0.24) | | | | | -1.181 | 0.24 |
| 16 | 0.193(0.16-0.22) | | | | | -1.809 | 0.22 |
| 17 | 0.173(0.14-0.20) | | | | | -1.181 | 0.20 |
| 18 | 0.154(0.12-0.18) | | | | | -1.809 | 0.18 |
| 19 | 0.127(0.10-0.16) | | | | | -1.181 | 0.16 |
| 20 | 0.106(0.08-0.14) | | | | | -1.809 | 0.14 |

| † The reference variable for drug fixed effects estimates is midazolam |
| --- |
| †† The reference variable for time fixed effects estimates is baseline |
| †† The reference variable for scale fixed effects estimates is Scale = 1 |
